# University students’ metacognitive awareness of reading strategies (MARS) in online reading and MARS’ role in their English reading comprehension

**DOI:** 10.1371/journal.pone.0313254

**Published:** 2024-11-08

**Authors:** Helta Anggia, Anita Habók

**Affiliations:** 1 Doctoral School of Education, University of Szeged, Szeged, Hungary; 2 Digital Learning Technologies Incubation Research Group, Institute of Education, University of Szeged, Szeged, Hungary; 3 MTA-SZTE Digital Learning Technologies Research Group, Szeged, Hungary; Universitas Nahdlatul Ulama Surabaya, INDONESIA

## Abstract

Investigation about metacognitive awareness of reading strategies (MARS) has mainly focused on paper-based reading rather than online reading. Gender, reading media preferences, and English proficiency levels (EPL) account for students’ differences in MARS. However, existing studies are still debating the predictive power of these variables on MARS. MARS was assumed to influence English reading comprehension even though research that firmly supports this assumption seems rare. Therefore, we examined Indonesian university students’ MARS in online reading activities using data from 1412 university students in Indonesia. They were categorized into gender, reading media preferences, and EPL. The One-Way MANOVA results showed students’ differences in MARS based on their gender, (F = 20.456, *p* < 0.05), reading media preferences, (F = 2.82, *p* < 0.05), and students’ EPL, (F = 5.988, *p* < 0.05). The multiple regression analysis showed that global, support, and problem-solving strategies were simultaneously associated with English reading comprehension scores. This study emphasizes the effect of the variables on students’ MARS differences and discloses the association between MARS and English reading comprehension.

## 1. Introduction

Readers’ awareness of reading strategies is known as metacognitive awareness of reading strategies (MARS) [1]. While most prior research focused on traditional paper-based reading (e.g., [2]), the impact of metacognitive awareness of reading strategies (MARS) in online reading contexts remains underexplored, especially in EFL contexts such as Indonesia [3]. This study addresses this gap by investigating how online reading strategies affect Indonesian EFL students’ comprehension.

Previous studies also focused on cognitive factors affecting MARS, such as the thinking process, information selection, and understanding evaluation in online reading that match the metacognitive skills [4]. When using metacognitive strategies in reading, readers determine the number of cognitive resources they need to judge their text comprehension [5]. In addition, highly proficient English readers are more likely to use problem-solving strategies rather than lower proficient readers who will use more support strategies [6–8]. All of these are the instance of cognitive factors’ role in the use of reading strategies. Studies investigating the involvement of non-cognitive factors affecting MARS in online reading have been very infrequent. Therefore, the theoretical gap that this study tried to fill in was the non-cognitive factors affecting MARS in online reading.

Some scholars believe that gender can impact metacognitive strategy use [9]. Previous studies have used several independent variables, such as gender, perceived English mastery, and many others, to show different levels of reading strategy use [1, 9]. In online reading, non-cognitive factors such as reading media and gender might be crucial in determining readers’ MARS. Moreover, scholars believe that MARS contributes to students’ English reading comprehension development [6–8].

To expand the current understanding, our study tried to see how online reading strategies were used in online EFL reading activities. In the case of Indonesian university students, most of them have low English language proficiency. As a result, they lack of English linguistics knowledge which is the most important requirement to apply metacognitive strategies when they read in English [10]. In the following section, we evaluate the main findings of previous empirical studies about MARS, especially cognitive and non-cognitive factors related to MARS and how MARS is associated with English reading comprehension.

## 2. Theoretical framework

### 2.1 Metacognitive awareness of reading strategies (MARS)

Sheorey and Mokhtari [1] categorized MARS into global, support, and problem-solving strategies. Global strategies are those created specifically to monitor the reading process, including previewing, and forecasting activities. On the other hand, support strategies are the fundamental categories that allow readers to interpret the text by utilizing a dictionary, taking notes, highlighting, or underlining textual content. Finally, problem-solving strategies enable readers to absorb difficult texts more successfully by deducing word meanings from context cues and visualizing text material.

### 2.2 The significance of gender in MARS

Taki and Soleimani [11] analyzed the online reading techniques of 15 male and 15 female Iranian EFL students. Survey of reading strategies (SORS) by [12] (2002) and online survey of reading strategies (OSORS) by Anderson [13] were adjusted. They found that male and female online reading techniques were identical. In a separate study, Öztürk and Aydogmus [2] explored the influence of gender on motivation and metacognitive reading strategies. The study employed a relational screening methodology, and 217 students from various classes and departments participated. Data was collected using the "Metacognitive Reading Strategies Measure" and the "Adult Reading Motivation Scale." They found no significant gender differences in the implementation of metacognitive strategies. Likewise, in a study that investigated the perceived use of online reading strategies by 94 Taiwanese EFL learners (40 males and 54 females), Chen [14] found that neither gender employs a different strategy. These three studies confirmed Sheorey’s and Mokhtari’s [1] claim that there is no gender difference in the use of cognitive reading strategies. However, a study conducted by Köse and Güneş [9] found a gender difference in the perceived use of strategies in reading where female students thought to use more reading strategies than the male students, thus indicating a significant difference in metacognitive reading strategies in reading based on gender. Although without much support, we assume that female students are more metacognitively aware of reading strategies than male students.

### 2.3 The significance of reading media preferences in MARS

Park et al. [15] emphasized the unique importance of hypermedia and computer applications for online reading. Their study analyzed the reading strategies employed by adult English language learners when reading online texts in hypermedia learning environments. The findings demonstrated that "blended" reading emphasized the various reaction patterns of the participants in their hypermedia learning contexts. Considering the uniqueness of the online learning environment that provides non-linear texts for readers [16], and what effect it may cause on the students’ navigation behavior in online reading [17], Anderson [13] adapted the survey of reading strategies (SORS) and established the online survey of reading strategies (OSORS). OSORS consisted of 38 items that belong to global, support, and problem-solving strategies. Each item revealed the unique characteristics of reading strategies used in online reading, and a wider possibility to apply reading strategies with help of technology. However, despite the increasing popularity of online reading, Ackerman’s and Goldsmith’s [18] reported the less accurate metacognitive prediction of online reading on English reading comprehension. In addition, Halamish and Elbaz [5] added up that students’ meta-comprehension in reading was not susceptible to any medium used in reading. Hence, the effect of reading media preference on students’ MARS is still debatable. However, we stood in our position that online reading has a positive impact on English reading comprehension. We maintained our stance that online reading positively impacts English reading comprehension by referring to empirical studies. For instance, Park et al. [15] emphasize the advantages of hypermedia in enhancing comprehension in online contexts. Moreover, research from Indonesia, such as Anggraini and Cahyono [3], supports that Indonesian students, despite linguistic challenges, benefit from online reading through better access to diverse reading materials and scaffolding technologies. Ackerman and Goldsmith [18] and Halamish and Elbaz [5], however, highlight the importance of metacognitive regulation in these environments. The distinctive features of online reading, such as non-linear texts and interactivity, foster greater cognitive engagement, thus enhancing comprehension.

### 2.4 The significance of English proficiency levels in MARS

MARS is strongly related with readers who have a high level of language competence, since they already possess the cognitive resources necessary to complete reading tasks [19, 20]. Metacognitive awareness and cognitive processes that enhance reading learning and problem-solving are important for readers. For example, metacognitive awareness or meta-comprehension is the essential capacity of native readers, enabling them to recognize their level of English reading comprehension and direct their learning to reach a desired understanding [21]. Typically, EFL readers have less metacognitive awareness than native readers [22]. Therefore, we assume that students with higher EPL are more metacognitively aware of reading strategies than tose with lower EPL.

### 2.5 The role of MARS in English reading comprehension

The majority of EFL readers utilize problem-solving strategies rather than global and supportive procedures [21]. When confronted with a book rich in lexical diversity, readers display their problem-solving abilities; they will reread the material, slow down, and concentrate more intently [19, 21]. The following review displays several empirical studies investigating students’ differences in MARS and the assumed relationship between MARS and English reading comprehension of university students across countries (Table 1). The review also indicates the most common instrument and sub-scales used for investigating MARS.

**Table 1:** Empirical studies of metacognitive strategy awareness.

Authors	Participants and context	Tool	Sub-scales	Key findings
Alkhateeb et al. [23]	127 university students; US 134 university students; in Qatar	MARSI	Global, Support, Problem-solving	The use of metacognitive reading strategies was prone to learners’ years of study, not gender.
Deliany and Cahyono [24]	53 undergraduate students; in Indonesia	MARSI-R	Global, Support, Problem-solving	Gender does not contribute to the differences in metacognitive reading strategy awareness and use.
Fisher [25]	92 EFL students; in Vietnam	MAI	Knowledge of cognition, regulation of cognition	Students’ metacognition does not constitute a whole because they cannot distinguish the reading strategies.
khellab et al. [6]	60 university students; in Libya	SORS	Global, Support, Problem-solving	Explicit instruction could help improve students’ metacognitive awareness and English reading comprehension.
Khreisat [26]	335 university students; in Saudi Arabia	MARSI-R	Global, Support, Problem-solving	Metacognitive reading strategies differ according to learners’ levels, not gender.
Ismail and Tawalbeh [27]	136 female university EFL students; in Saudi Arabia	SORS	Global, Support, Problem-solving	Metacognitive reading strategy training improves reading skills.
Al-Mekhlafi [28]	74 undergraduate EFL learners; in Oman	MARSI and SORS	Global, Support, Problem-solving	Metacognitive reading strategy awareness is not prone to students’ levels.
Muhid et al. [7]	50 secondary school students; Indonesia	MSQ	Planning, Monitoring, Evaluating	Metacognitive strategies affect English reading comprehension, and selective attention is the highest in use.
Thuy [29]	81 TESOL postgraduate students; Vietnam	SORS	Global, Support, Problem-solving	Some of the metacognitive strategies were often neglected by the students when reading.
Villanueva [8]	446 college students; the Philippine	MARI	Global, Support, Problem-solving	Problem-solving strategy contributes most to English reading comprehension achievement.

## 3. Research aims

As mentioned above, previous studies have indicated that gender, reading media preferences, and EPL have an effect on students’ MARS. Meanwhile, MARS’ sub-scales (Global, Support, and Problem-Solving Strategies) were associated with English reading comprehension.

H1: Female students are expected to have more metacognitive awareness of reading strategies than the male students.
H2: Online reading media is expected to influence students’ metacognitive awareness of reading strategies than paper-based reading media.
H3: Students with higher English proficiency level are more metacognitively aware of reading strategies than are the lower English proficiency level students.
H4: MARS are expected to be positively associated with English reading comprehension.

Therefore, we formulated the following research questions:

RQ1: Are there any significant differences in the students’ MARS in terms of gender, reading media preference, and English proficiency levels?
RQ2: Does a structural relationship exist between global, support, and problem-solving strategies with English reading comprehension scores among EFL university students in Indonesia?

## 4. Method

### 4.1 Participants

This study was a cross-sectional study that gathered data from 1412 university students. We selected 33 classes randomly from thirteen universities in Indonesia (Table 2). The students received basic English language classes in their first two semesters at university and claimed to have actively read in English in their daily life. The data of the students’ self-report were collected using the Google Form. Participants obtained informed written consent at the start of the study. Participation in the research was only possible after written informed consent. The recruitment period of the research lasted from 30/March/2022 to 30/September/2022.

**Table 2:** Demographics of participants.

Characteristics	Number of Sample	Percentage (%)
Gender		
Female	1001	70.9
Male	411	29.1
Reading Media Preference		
Paper-based	159	11.3
Online-based	404	28.6
Blended	849	60
English Proficiency Levels		
A1	401	28.4
A2	675	47.8
B1	73	5.2
B2	208	14.7
C1	55	3.7

### 4.2 Materials

The study used online questionnaires and English reading comprehension tests using the Google Forms platform. *Metacognitive Awareness of Reading Strategies*. The self-reported questionnaire about the OSORS consisting of 38 five-Likert-scale items [13] was used (Cronbach’s α = 0.976). The questionnaire consisted of items on global strategies (e.g., I have a purpose in mind when I read online), problem-solving strategies (e.g., I read slowly and carefully to make sure I understand what I am reading online.), and support reading strategies (e.g., I take notes while reading online to help me understand what I read). Each item was rated using a four-point response scale, namely, *never* = 0, *once in a while* = 1, *sometimes* = 2, *normally* = 3, and *always* = 4. The Confirmatory Factor Analysis indicated the loading factor of each item in OSORS, > 0.40. The model fit of the data showed excellent goodness of fit, comparative fit index (CFI) = 0.993, Tucker–Lewis index (TLI) = 0.993, and root mean square error of approximation (RMSEA) = 0.083 (Table 3). It means that OSORS was considered valid in the Indonesian university context.

**Table 3:** Model fit of OSORS.

Chi-square	*df*	*p*<	CFI	TLI	RMSEA	Estimator
7124.520	662	0.001	0.993	0.993	0.083	DWLS

Note. df = degree of freedom; CFI = Comparative fit index; TLI = Tukey Lewis index; RMSEA = Root mean square error of approximation; DWLS = Diagonally weighted least squares

#### 4.2.1 English reading comprehension test

A 20-question multiple-choice test on English reading comprehension adopted from the ReadTheory.org reading worksheet Dao [30] was given to the students (Cronbach’s α = 0.70). The reading test consisted of five short passages with four questions following each passage. The questions were: main idea (e.g., what is the main idea of the passage?), detailed (e.g., it is easier to see moonbows in rural areas because.), inferring (e.g., from the passage we can infer that.), and vocabulary questions (e.g., the word "tallest" in the second line of the first paragraph is closest in meaning to). For each correctly answered item, a score of 1 was given, while the incorrectly answered item was given 0.

### 4.3 Procedure

The Institutional Review Board (IRB) of the Doctoral School of Education, University of Szeged authorized the research and the full ethics statement was provided by the Doctoral School of Education, University of Szeged. 1412 participants gave their informed consent, signifying that they agreed to participate in the research. Then, we talked to the teachers at the targeted universities and asked if they could tell the students how to do the questionnaire and reading test. The teachers spent a few minutes explaining to their students how to fill out the given questionnaire and do the reading test.

### 4.4 Data analysis

Using SPSS 25, the correlation among the variables was analyzed using Pearson’s Product-Moment Correlation [31], and the One-Way MANOVA analysis was employed to see the students’ differences in MARS based on gender, reading media preference, and EPL [32]. To visualize the results of the difference analyses in this study, we employed a pirate plot produced by the Yarrr package in R [33]. Finally, the structural relationship between the sub-scales of MARS and English reading comprehension was analyzed using a multiple regression model analysis [34].

## 5. Results

### 5.1 Correlation of categorical, latent variables and English reading comprehension

The study carried out a correlational analysis to assess the relationships between the variables involved. The variables were gender, reading media preference, EPL, global strategies, support strategies, problem-solving strategies, and English reading comprehension. Nearly all variables were significantly correlated, apart from reading media preference with support strategies (r(1412) = 0.046, p > 0.001), reading media preference with problem-solving strategies (r(1412) = 0.050, p > 0.001), reading media preference with English reading comprehension (r(1412) = 0.039, p > 0.001), and gender with English reading comprehension (r(1412) = -0.037, p > 0.001). The most notably correlated variables between either one of MARS (global, support, and problem-solving strategies) and other variables were EPL and problem-solving strategies (r(1412) = 0.203, p < 0.001). The remaining variables were also significantly correlated, although the strength of the correlation was weaker.This correlation suggested that we could assume an association between the three variables and English reading comprehension (Table 4).

**Table 4:** Correlation among variables.

	G	RM	EPL	GLOB	SUP	PROBL	RC
Gender	1						
Reading media preference	- 0.084**	1					
EPL	-0.174**	0.074**	1				
Global strategies	-0.103**	0.065*	0.200**	1			
Support strategies	-0.166**	0.046	0.168**	0.910**	1		
Problem-solving strategies	-0.130**	0.050	0.203**	0.930**	0.910**	1	
Reading comprehension	-0.037	0.039	0.178**	0.107**	0.119**	0.179**	1

**. Correlation is significant at the 0.01 level (2-tailed).

*. Correlation is significant at the 0.05 level (2-tailed).

### 5.2 Metacognitive awareness of reading strategies differences

One-Way MANOVA test was conducted to determine the students’ MARS differences based on gender, reading media preference, and EPL. The results revealed a statistically significant difference among the three strategies (Table 5). There was a significant difference in MARS scores between male and female students, F (4, 1404) = 20.456, p < 0.05; Wilk’s = 0.958; η^2^ = 0.042, among reading media preferences, F (4, 1404) = 2.82, p < 0.05; Wilk’s = 0.988; η^2^ = 0.006, and among students’ EPL, F (4, 1404) = 5.988, p < 0.05; Wilk’s = 0.951, η^2^ = 0.017. Consequently, the alternative hypotheses (Hypotheses 1–3) were supported, and we concluded that there were statistically significant differences in the MARS (global, support, and problem-solving) levels of students across gender, reading media preference, and EPL. However, the partial eta squared (η^2^) results of each variable showed low effect size (Table 5).

**Table 5:** Results for One-Way MANOVA for all dependent variables (global, support, and problem-solving strategies).

Source	Wilks’ Λ	*F*	Hypothesis df	Error df	Sig.	η^2^
Gender	0.958	20.456	3.000	1408.000	0.000	0.042
Reading media preference	0.012	2.823	6.000	2814.000	0.010	0.006
EPL	0.951	5.988	12.000	3709.635	0.000	0.017

Note. The p-value was significant < 0.05

**Fig 1:**
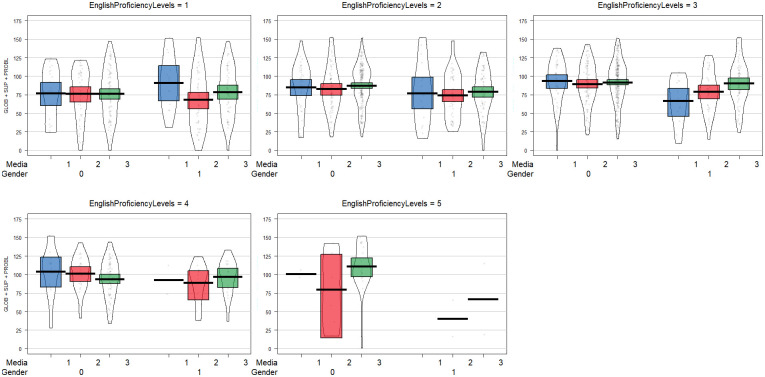
Metacognitive awareness of reading strategy means differences across gender, reading media preference, and EPL. Note. **Black color bars** = mean scores; **Band** = Inference around the mean, either a Bayesian Highest Density Interval (HDI), or a Confidence Interval (CI); **Points** = Raw data; **Bean** = Smoothed density curve showing the full data distribution;**Media** (1 = Paper-Based Reading Media Preference; 2 = Online-Based Reading Media Preference; 3 = Blended Reading Media Preference); **Gender** (0 = Female; 1 = Male).

Fig 1 shows the raw data, descriptive, and inferential statistics of the university students’ MARS according to their gender, reading media preference preferences, and EPL. Across the EPL, female C1 students who preferred blended reading media have the highest mean scores of MARS relative to the other students of all EPL groups. On the contrary, the male C1 students are among the lowest in terms of their MARS mean scores. That is due to the small number of male C1 students in our sample. Except for the group EPL 1/A1, overall, female students of the other groups, given their reading media preferences, outperformed the male students in their MARS. As can be seen from bands in the pirate-plot, all the students of every EPL group who preferred to read on paper had a wider variation in their MARS relative to other reading media preferences. In most groups, if not all, students who blended paper and online reading outperformed other students who used either one paper or online-based reading.

Regarding the reading media preference, post hoc multiple comparisons utilizing the Tukey HSD test revealed that mean scores for global strategies, support strategies, and problem-solving strategies were statistically significant in mean comparisons between blended reading media preference and online reading media preference (p < 0.05), with blended reading media preference evoking a greater metacognitive awareness of reading strategies among students. Students who utilize both paper-based and online reading materials are more metacognitively aware of reading strategies than those who use exclusively online reading materials.

Regarding EPL, post hoc multiple comparisons utilizing the Tukey HSD test revealed that mean scores for global strategies were statistically significant between the levels (p < 0.05), with the exception of comparisons between B1 and B2 proficiency levels (p > 0.05), B1 and C1 levels (p > 0.05), and B2 and C1 levels (p > 0.05). The mean scores for support strategies were statistically significant between levels (p < 0.05) with the exception of comparisons between levels A1 and A2 (p > 0.05). A2 and C1 were significantly different (p < 0.05), as were B1 and B2, B1 and C1, and B2 and C1. The mean scores for problem-solving strategies were statistically significant between levels (p < 0.05), with the exception of comparisons between B1 and B2 (p > 0.05). B1 and C1 were significant (p < 0.05), as were B2 and C1. Students with greater levels of English proficiency, such as B1, B2, and C1, differ considerably from students with lower levels of English proficiency, such as A1 and A2, in terms of their awareness of reading strategies.

### 5.3 MARS and English reading comprehension

A multiple regression analysis was performed to see the association between the three aspects of the MARS and the students’ English reading comprehension scores (Table 6). All the independent variables (global, support, and problem-solving strategies) simultaneously influenced the dependent variable (students’ English reading comprehension scores), R^2^ = 0.060, *F* (1411) = 30.136, *p* = 0.000. This means that all the variance of the independent variables influenced the dependent variable by 6%. Therefore, this finding supported our hypothesis that there was a positive direct structural relationship between the students’ MARS and their English reading comprehension scores. However, each strategy, partially significantly influenced the students’ English reading comprehension scores except for support strategy *t* (2) = -1.648, *p* = 0.100.

**Table 6:** Coefficients table of multiple regression analysis.

Model summary					
Multiple R	0.246				
R^2^	0.060				
Adjusted R^2^	0.058				
Standard error	3.55220				
Analysis of variance					
	Sum of square	DF	Mean square	F	Sig.
Regression	1140.788	3	380.263	30.136	0.000
Residual	17766.325	1408	12.618		
Total	18907.113	1411			
Coefficients: variables included in the equation					
	Unstandardized Coefficients		Standardized coefficients		
	B	Std error	Beta	*t*	Sig.
Constant	8.285	0.273		30.339	0.000
Global	-0.095	0.019	-0.387	-5.011	0.000
Support	-0.052	0.032	-0.113	-1.648	0.100
Problem-solving	0.237	0.029	0.642	8.313	0.000

## 6. Discussion

The finding that reading media preference was not significantly correlated with support strategies (r(1412) = 0.046, p > 0.001) may suggest that the medium used for reading (whether online or paper-based) does not influence the use of support strategies like note-taking or using a dictionary. This supports the notion that students apply support strategies in a similar manner across different media [5, 18]. The weak correlation between reading media preference and problem-solving strategies (r(1412) = 0.050, p > 0.001) suggests that problem-solving strategies, such as rereading difficult text or deducing word meanings from context, are not affected by whether students read online or on paper. This aligns with previous research indicating that these cognitive processes are not medium-specific [5]. The non-significant relationship between reading media preference and English reading comprehension (r(1412) = 0.039, p > 0.001) implies that reading comprehension is not dependent on the reading medium. This supports Ackerman and Goldsmith [18] findings that comprehension is more related to cognitive factors like metacognitive awareness rather than the medium of reading. The lack of a significant correlation between gender and English reading comprehension (r(1412) = -0.037, p > 0.001) suggests that gender does not play a substantial role in comprehension abilities in this context, which is consistent with studies like Sheorey and Mokhtari [1], who found no significant gender differences in reading comprehension abilities.

The novelty of this study lies in our analysis of university students’ MARS in online reading, which comprises global, support, and problem-solving strategies that have been rarely discussed, especially in the context of English as a foreign languages [3]. In addition, most previously conducted studies of EFL reading focused on paper-based reading strategies [2, 9]. Moreover, our findings, especially regarding the effect of gender, and students’ reading media preference on MARS gave us a new remarkable insights that non-cognitive factors also determine students’ MARS differences.

### 6.1 RQ1

*First*, the gender difference applied to all the strategies, global, support, and problem-solving strategies as in the study of Sheorey and Mokhtari [1]. It was in contrast with previous studies by Öztürk and Aydogmus [2]; Chen [14]; Sheorey and Mokhtari [1], Taki and Soleimani [11], Alkhateeb et al. [23], Deliany and Cahyono [24], and Khreisat [26] that gender has no significant effect on metacognitive awareness of reading strategies in English reading as a foreign language, and there is no psychological aspect of gender that might cause students’ differences in metacognitive awareness of reading strategies. The assumption behind this finding stems from multiple studies suggesting that cognitive processes involved in reading strategies transcend gender-specific psychological differences. Chen [14] and Öztürk and Aydogmus [2] observed no significant differences between males and females in the use of metacognitive strategies, likely because reading strategy awareness is shaped more by individual cognitive engagement and proficiency than by gendered psychological traits. While some studies (e.g., [9]) note minor gender-related distinctions, these are generally considered negligible in the broader context of language learning. On the other hand, our finding was in line with Köse’s & Güneş’ [9] who found a significant gender difference in students’ metacognitive awareness of reading strategies. Our findings highlighted the significance of gender differences in foreign language reading, confirming our first hypothesis.

*Second*, as for the reading media preferences, we categorized them into three types of reading media preference. The first category was paper-based reading media preference by which the students relatively preferred reading English texts using printed materials such as books, magazines, etc to online reading media. The second was online materials by which the students could access online reading materials using their screen reading devices such as personal computers, mobile, and tablets. The last category was “blended” reading which combined reading on printed materials and online reading. Blended reading was quite popular since the students might sometimes mix their reading media depending on their actual preference. The findings of the current study showed that the variance was significant in the paper-based, online reading media, and blended reading mean scores, corroborating our second hypothesis. The finding of the multivariate analysis of variance and the result of the Multicomparison Tukey HSD post hoc test indicated that the students who preferred using online reading media alone were lack in the use of metacognitive reading strategies when reading in English. This fact was interesting since it contrasted the unique characteristics of online reading media that provide non-linear texts for the readers [16], and the influence of online reading on readers’ navigation behavior in reading [17]. The finding was in line with previous studies that stated that online reading had less predictive power on students’ effort in doing an English reading comprehension task [5, 18].

On the other hand, the students who preferred to mix the use of paper-based and online reading media were most aware of the use of reading strategies. It related to the notion that blended reading triggered students’ preference in the hypermedia context [15]. From the findings, we could understand that paper-based reading should not be at all neglected. It means that readers may combine printed and online reading strategies in English reading by combining the use of paper-based reading and online-based reading media to enhance their comprehension. This strategy has been widely adopted by flipped classroom activities. Combining printed and online reading is adaptive to the students’ actual preference when reading multitexts, as seen by the variation in students’ metacognitive awareness of reading strategies scores based on the reading mediums they employed. Our results showed significant differences in MARS based on reading preferences, highlighting the nuanced interaction between English proficiency levels and metacognitive strategy use. For example, students using blended reading strategies exhibited higher awareness than those using only online media, suggesting that combining paper-based and digital reading facilitates deeper engagement [15]. This underscores the importance of hybrid instructional models in improving MARS.

Additional research can be undertaken to determine when children prefer to read on paper versus on a screen. The students’ metacognitive awareness of reading strategies difference based on reading media preference applied to all three strategies, global, support, and problem-solving strategies as proposed by [1].

Eventually, despite the difference in metacognitive awareness of reading strategies between different use of reading media preference being very small according to the effect size results (Table 5), we still believed that the practical significance of mixed reading media preference use in the classroom can be seen after we conduct an experimental study on English reading. Therefore, conducting an experimental study on various reading media preference is strongly recommended.

*Third*, the students’ metacognitive awareness of reading strategies differences based on English proficiency levels applied to all three strategies, global, support, and problem-solving strategies as proposed by [1], confirming our third hypothesis that the higher the English proficiency level, the more students become metacognitively aware of reading strategies. Our findings supported the previous studies by [19–21] that English proficiency levels influence the metacognitive awareness of reading strategies. With either a high English proficiency level or nativeness of English, readers can put more attention to the use of certain reading strategies for a given reading challenge rather than having to struggle with linguistics challenge [10]. The multiple comparisons Tukey HSD post hoc test addressed the common substantial variation in the mean scores between the students’ English proficiency levels. In general, students with a greater level of English proficiency were more able to examine and monitor (implementing global strategies) their comprehension when reading online than students with a lower level of English proficiency. In contrast, each group of students with varying degrees of English ability differed just a little in their awareness of support strategies. The insignificance of EPL groups on support strategies corroborated our conclusion from students’ MARS difference based on reading media preference that online reading media preference alone cannot predict students’ metacognitive awareness of reading strategies. In online reading, there are resources to assist students in using support strategies, but students cannot rely solely on online reading to execute support strategies. This is the reason why many students opted to use both paper-based and online reading media to help them implement support strategies in reading. The problem-solving strategies of students with higher English proficiency levels, such as C1, B2, and B1, differed significantly from those of students with lower English proficiency levels. It confirmed that students who combined paper-based and online reading media gained a greater metacognitive awareness of reading strategies.

The results showed significant differences in MARS based on gender, reading preferences, and English proficiency level, highlighting the complex interactions of these factors with students’ reading strategies. This result is significant for English teachers to consider the above three aspects in training their students’ metacognitive awareness in reading strategies.

### 6.2 RQ2

The next aim of the study was to see the association direction between metacognitive awareness of reading strategies and the students’ English reading comprehension. As the findings reported a simultaneous significant effect of metacognitive awareness of reading strategies on English reading comprehension, confirming our fourth hypothesis, it means that the results of the study are in line with Ismail and Tawalbeh [27]; khellab et al. [6]; Muhid et al. [7]; Villanueva [8] that metacognitive awareness of reading strategies have an association with English reading comprehension. Using the same sub-scales in their instruments as in our current study, they found that metacognitive awareness of reading strategies can be enhanced through specific training. Therefore, metacognitive awareness of reading strategies facilitates metacognitive awareness of reading strategies training with English reading comprehension scores. The findings also confirmed the previous studies that the most used strategies in reading were problem-solving [19, 21]. More practice in problem-solving strategies will make the students become efficient readers [25]. Interestingly, support strategies in online reading negatively influence English reading comprehension. This finding could have related to our answer to RQ 1 that the students did not differ in implementing support strategies based on both reading media preferences and English proficiency levels. The finding indicated that online reading does not alter students’ behavior in using support strategies in reading. It is quite surprising considering the fact that online reading, with the help of technology is supposed to provide the students with a lot of options of reading features to support students’ reading comprehension.

## 7. Limitation

Despite the fact that the findings provide empirical support for our theoretical hypothesis regarding MARS differences among students and a suspected association between MARS and English reading comprehension, the effect size and R^2^ values are quite low. Due to the cross-sectional nature of our data collection method, the results of the regression coefficient cannot be interpreted as a causal relationship. The causal relationship between metacognitive awareness of reading strategies (MARS) and English reading comprehension requires longitudinal research. In addition to MARS, we believe there are a multitude of factors that predict English reading comprehension that are worth investigating.

## 8. Conclusion and implication

The current study yields some noteworthy conclusions on the metacognitive awareness of reading strategies among Indonesian university students when reading in English. *First*, in the English as a foreign language environment, gender disparities in metacognitive awareness of reading strategies exist among university students in Indonesia. *Second*, the predictive power of online reading preference on students’ metacognitive awareness is less relative to paper-based and blended reading media preference. *Third*, students with higher levels of English employ metacognitive reading strategies more frequently than those with lower levels of English. *Fourth*, Students’ use of global and problem-solving strategies influences English reading comprehension. On the other hand, support strategies negatively influence English reading comprehension.

The findings of this study are significant for university teachers in Indonesia to address the metacognitive awareness of reading strategies differences of their students based on gender, reading media preference, and English proficiency levels to assist their students in becoming more strategic readers. Especially for struggling readers, constant practice of using problem-solving strategies could be the fastest solution to comprehend English reading text well.

These findings emphasize the need to account for individual differences when designing EFL reading programs. For instance, instructional strategies in online reading environments should foster metacognitive strategy use among less proficient readers by encouraging blended reading approaches. The broader implication for educational practices is to balance digital and traditional methods to support comprehensive skill development.

## Supporting information

S1 Data (XLS)
